# A novel pyroptosis scoring model was associated with the prognosis and immune microenvironment of esophageal squamous cell carcinoma

**DOI:** 10.3389/fgene.2022.1034606

**Published:** 2023-01-04

**Authors:** Zhan-Fei Zhang

**Affiliations:** ^1^ Department of Cardiothoracic Surgery, Zhongshan People’s Hospital, Zhongshan, China; ^2^ State Key Laboratory of Oncology in South China, Collaborative Innovation Center for Cancer Medicine, Department of Experimental Research, Sun Yat-Sen University Cancer Center, Guangzhou, China

**Keywords:** pyroptosis scoring model, prognosis, immune microenvironment, esophageal squamous cell carcinoma, biomarker

## Abstract

The phenotype of pyroptosis has been extensively studied in a variety of tumors, but the relationship between pyroptosis and esophageal squamous cell carcinoma (ESCC) remains unclear. Here, 22 pyroptosis genes were downloaded from the website of Gene Set Enrichment Analysis (GSEA), 79 esophageal squamous cell carcinoma samples and GSE53625 containing 179 pairs of esophageal squamous cell carcinoma samples were collected from the Cancer Genome Atlas (TCGA) and the Gene Expression Omnibus (GEO), respectively. Then, pyroptosis subtypes of esophageal squamous cell carcinoma were obtained by cluster analysis according to the expression difference of pyroptosis genes, and a pyroptosis scoring model was constructed by the pyroptosis-related genes screened from different pyroptosis subtypes. Time-dependent receiver operator characteristic (timeROC) curves and the area under the curve (AUC) values were used to evaluate the prognostic predictive accuracy of the pyroptosis scoring model. Kaplan-Meier method with log-rank test were conducted to analyze the impact of the pyroptosis scoring model on overall survival (OS) of patients with esophageal squamous cell carcinoma. Nomogram models and calibration curves were used to further confirm the effect of the pyroptosis scoring model on prognosis. Meanwhile, CIBERSORTx and ESTIMATE algorithm were applied to calculate the influence of the pyroptosis scoring model on esophageal squamous cell carcinoma immune microenvironment. Our findings revealed that the pyroptosis scoring model established by the pyroptosis-related genes was associated with the prognosis and immune microenvironment of esophageal squamous cell carcinoma, which can be used as a biomarker to predict the prognosis and act as a potential target for the treatment of esophageal squamous cell carcinoma.

## 1 Introduction

Esophageal cancer (EC) includes esophageal squamous cell carcinoma (ESCC) and esophageal adenocarcinoma (EAC), it is one of the deadliest cancers in the world (Lagergren et al., 2017; Smyth et al., 2017). China has a high prevalence of EC that accounts for more than 50% of the global morbidity and mortality, and over 90% of patients with EC in China were ESCC (Abnet et al., 2018; Cao et al., 2021). However, due to the low early diagnosis rate, prone to invasion and metastasis, and insensitivity to radiotherapy and chemotherapy, ESCC patients still have a low 5-year overall survival (OS) rate despite multidisciplinary treatment including surgery, chemotherapy and radiotherapy (Hirano and Kato, 2019; Harada et al., 2020; Yang et al., 2020). Therefore, there is an urgent need for a new strategy to improve the prognosis of ESCC patients.

Pyroptosis is the programmed cell necrosis mediated by gasdermins (Hou et al., 2021), which is an important innate immune response in the body and plays an important role in fighting infection (Xia et al., 2019; Li et al., 2021a). At present, it is known that pyroptosis is closely related to a variety of diseases, and is widely involved in the occurrence and development of infectious diseases (Zhao et al., 2022), nervous system-related diseases (Jin et al., 2022), atherosclerotic diseases (Guo et al., 2022), tumors (Deng et al., 2022; Liu et al., 2022; Niu et al., 2022) and other diseases (Al Mamun et al., 2022; Wen et al., 2022). Pyroptosis plays a dual role in the occurrence and development of tumors (Yu et al., 2021), on one hand, as an innate immune mechanism, pyroptosis can inhibit the occurrence and development of tumors, on the other hand, as a way of pro-inflammatory cell death, pyroptosis provides a suitable microenvironment for tumor growth (Du et al., 2021). Pyroptosis is divided into classical pathways and non-classical pathways. Inflammasomes, gasdermin proteins, and pro-inflammatory cytokines are all key components of the pyroptosis pathways. Various components of the pyroptosis pathways can be regulated by a variety of cell signaling pathways. Various components of the pyroptosis pathways can regulate cell morphology, proliferation, invasion, migration, chemotherapy resistance and other malignant phenotypes through a variety of cell signal pathways, thus affecting tumor progression, and may be related to the prognosis of patients (Tan et al., 2021).

At present, the research on pyroptosis and EC mainly focuses on promoting the sensitivity of EC cells to chemoradiotherapy through inducing pyroptosis of EC cells *via* various drugs or techniques (Wu et al., 2019; Fang et al., 2020; Li et al., 2021b; Li et al., 2022b), but there are still few studies on pyroptosis and ESCC, the roles and mechanisms of pyroptosis in ESCC are far from clear (Jiang et al., 2021), and more studies are needed to elucidate the relationship between ESCC and the pyroptosis phenotype.

This study found that there were different pyroptosis subtypes in ESCC, and the pyroptosis scoring model constructed by pyroptosis-related genes was related to the prognosis and immune microenvironment of ESCC. This study improved our understanding of ESCC and provided a new insight for the treatment of ESCC. The development of drugs targeting pyroptosis may be a new strategy for the treatment of ESCC, which has a good therapeutic prospect.

## 2 Materials and methods

### 2.1 Data acquisition

The per million reads (TPM) format gene expression profile data of ESCC was downloaded from the Cancer Genome Atlas (TCGA, https://portal.gdc.cancer.gov/) using the TCGAbiolinks (Colaprico et al., 2016) R package, clinical information of ESCC was downloaded by GDC software, and 79 samples were finally obtained by matching gene expression profile with clinical data. GSE53625, the largest ESCC dataset in the Gene Expression Omnibus (GEO, https://www.ncbi.nlm.nih.gov/gds) so far, contains 358 samples, including 179 tumor samples and 179 matched normal samples, all of which contain clinical information, and was downloaded by the GEOquery (Davis and Meltzer, 2007) R package. The 22 pyroptosis genes were collected from the official website of Gene Set Enrichment Analysis (GSEA, https://www.gsea-msigdb.org/gsea/index.jsp), including DHX9, GSDME, NLRP6, ELANE, NLRP1, GSDMA, GZMA, GZMB, NLRP9, NAIP, APIP, TREM2, GSDMB, GSDMC, NLRC4, GSDMD, ZBP1, CASP1, CASP4, CASP6, CASP8, AIM2. This study was in compliance with the published guidelines of TCGA and GEO, thus, ethical approval and informed consent of the patients were not required.

### 2.2 The expression difference of pyroptosis genes

According to the tissue source, the samples in the GSE53625 were divided into the normal group (*n* = 179) and the tumor group (*n* = 179). Next, the expression differences of 20 pyroptosis genes expressed in GSE53625 were showed by boxplots using the ggplot2 R package.

### 2.3 Pyroptosis subtypes analysis and differential expression analysis

According to the expression difference of pyroptosis genes in TCGA-ESCC, the samples were clustered into two clusters by ConsensusClusterPlus (Wilkerson and Hayes, 2010) R package. The differentially expressed genes (DEGs) of the two subtypes were obtained by differential expression analysis using the DESeq2 (Love et al., 2014) R package. The genes with logFC > 1 and adj.*p* < .05 were upregulated DEGs, and the genes with logFC < −1 and adj.*p* < .05 were downregulated DEGs, a heatmap of DEGs were displayed by pheatmap R package, and the DEGs were used for subsequent analysis.

### 2.4 Functional enrichment analysis

The clusterProfiler (Yu et al., 2012) R package was used to perform Gene Ontology (GO) functional analysis and Kyoto Encyclopedia of Genes and Genomes (KEGG) pathway analysis based on DEGs, GO functional analysis including biological process (BP), cellular composition (CC) and molecular function (MF) analysis, and a cut-off value of false discovery rate (FDR) < .05 was considered statistically significant. GSEA was also conducted by clusterProfiler R package, the “c2.cp.kegg.v6.2.symbols” gene set was downloaded as the reference gene set from MSigDB database (https://www.gsea-msigdb.org/gsea/msigdb/index.jsp), and FDR < .25 was considered significantly enriched. GSEA and Gene Set Variation Analysis (GSVA) were performed by the GSVA (Hnzelmann et al., 2013) R package, “c2.cp.kegg.v6.2.symbols” and “h. all.v7.0.symbols” gene set were used as references.

### 2.5 Construction and evaluation of pyroptosis scoring model

We combined the TCGA-ESCC and GSE53625 data after the batch effect was removed by the SVA R package. Next, we randomly divided the merged dataset into a training set and a validation set with a ratio of 1:1. Then, univariate Cox regression analysis was performed to calculate the association between the DEGs and OS, and the genes with *p* < .05 were selected for the least absolute shrinkage and selection operator (Lasso) in the training set by the glmnet R package. The pyroptosis scoring model was constructed based on the selected pyroptosis-related genes, and the patients were divided into high pyrop_group and low pyrop_group according to the median of the pyroptosis score. Time-dependent receiver operating characteristic (timeROC) curves were applied to assess the prognostic predictive accuracy at 1-year, 2-year, 3-year and 5-year by the timeROC R package. In addition, Kaplan-Meier method with log-rank test were conducted to evaluate the impact of the pyroptosis scoring model on the OS of ESCC. Furthermore, a nomogram included the pyroptosis score was constructed to predictive the OS of ESCC patients, and Calibration curves were also employed to observe the fitting of the actual survival probability and the predicted survival probability at 1-year, 3-year, and 5-year.

### 2.6 Correlation analysis between pyroptosis scoring and immune cell infiltration

We uploaded the training set and validation set to the CIBERSORTx (https://cibersortx.stanford.edu/), respectively, LM22 was used as the reference dataset, and the distribution of 22 types of immune cell infiltration in each sample was displayed by histograms using ggplot2 R package. The ESTIMATE algorithm was used to evaluate the effect of pyroptosis scoring on immune cell infiltration in ESCC.

### 2.7 Statistical analysis

All the calculations and statistical analyses were performed by R (version 4.0.2). For comparison of continuous variables between two groups, the statistical significance of normally distributed variables was estimated by independent Student’s *t*-test, and differences between non-normally distributed variables were analyzed by Mann-Whitney U test. The timeROC R package was used to draw the timeROC curves and calculate the area under the curve (AUC) values. Kaplan-Meier method with log-rank test was used to survival analysis. In all tests, *p* < .05 was considered statistically significant.

## 3 Results

### 3.1 The expression differences of pyroptosis genes in ESCC

**FIGURE 1:**
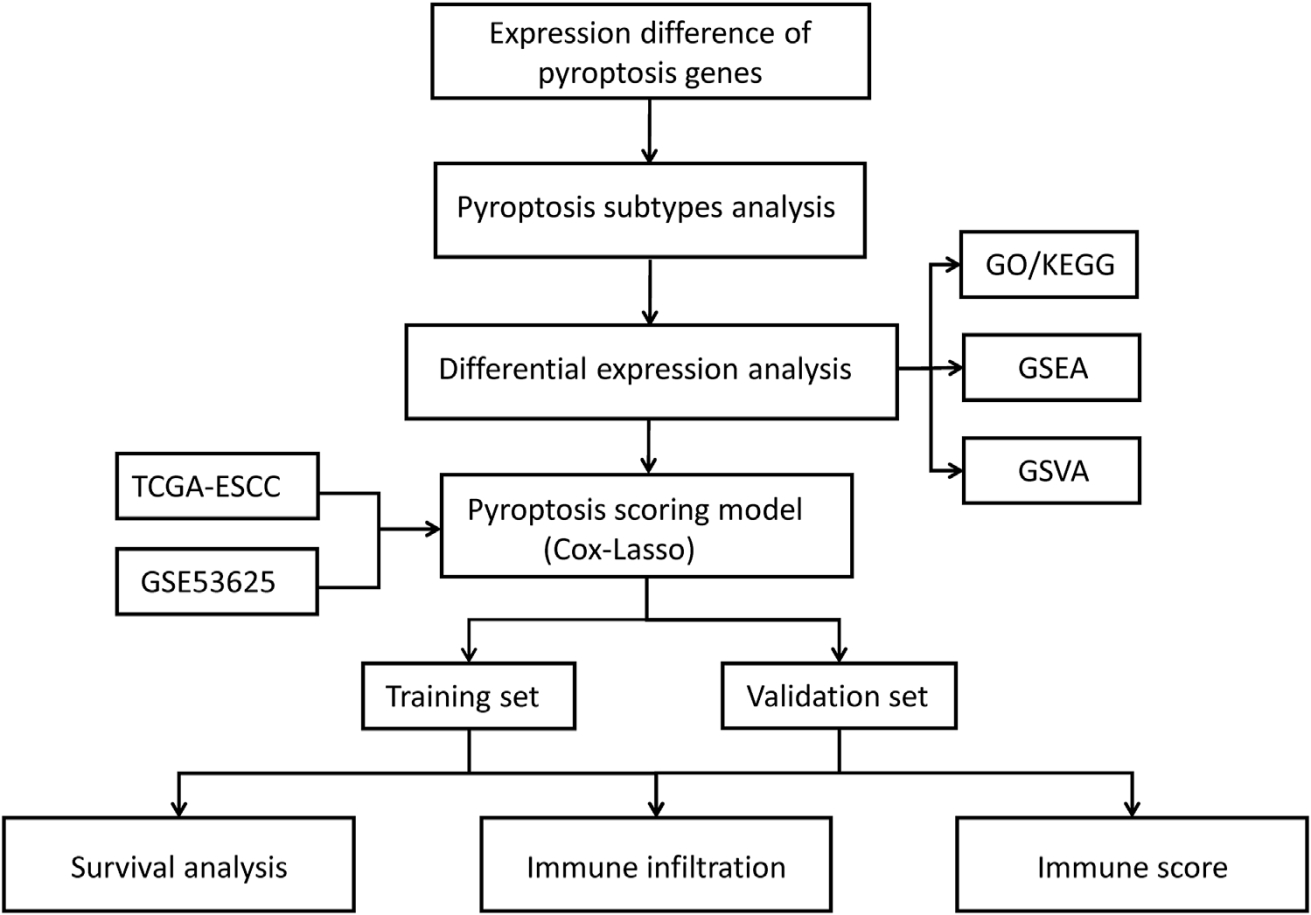
Flow chart of this study.

**FIGURE 2:**
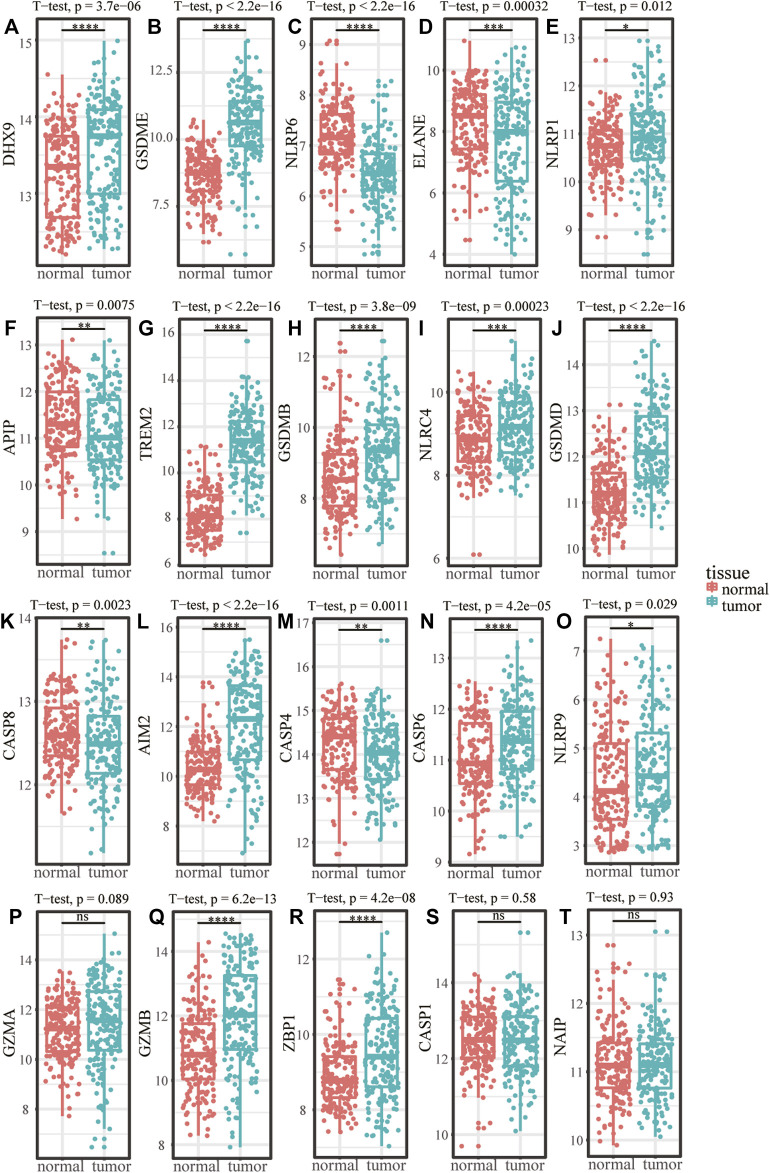
The expression difference of pyroptosis genes in ESCC. **(A–T)** Expression difference of pyroptosis genes between normal samples and tumor samples in GSE53625. *p < .05; **p < .01; ***p < .001; ****p < .0001; ns, *p* > .05.

The flow chart of this research was shown in Figure 1. Among the 22 pyroptosis genes, 20 were matched in GSE53625, and the expression differences of the 20 pyroptosis genes in tumor and normal tissues were analyzed. The results showed that compared with the normal samples, DHX9, GSDME, NLRP1, GZMB, NLRP9, TREM2, GSDMB, NLRC4, GSDMD, ZBP1, CASP6, and AIM2 were higher expressed in tumor samples, whereas NLRP6, ELANE, APIP, CASP4, and CASP8 were lower expressed in tumor samples (Figures 2A–T).

### 3.2 Subtypes analysis of pyroptosis genes in ESCC

**FIGURE 3:**
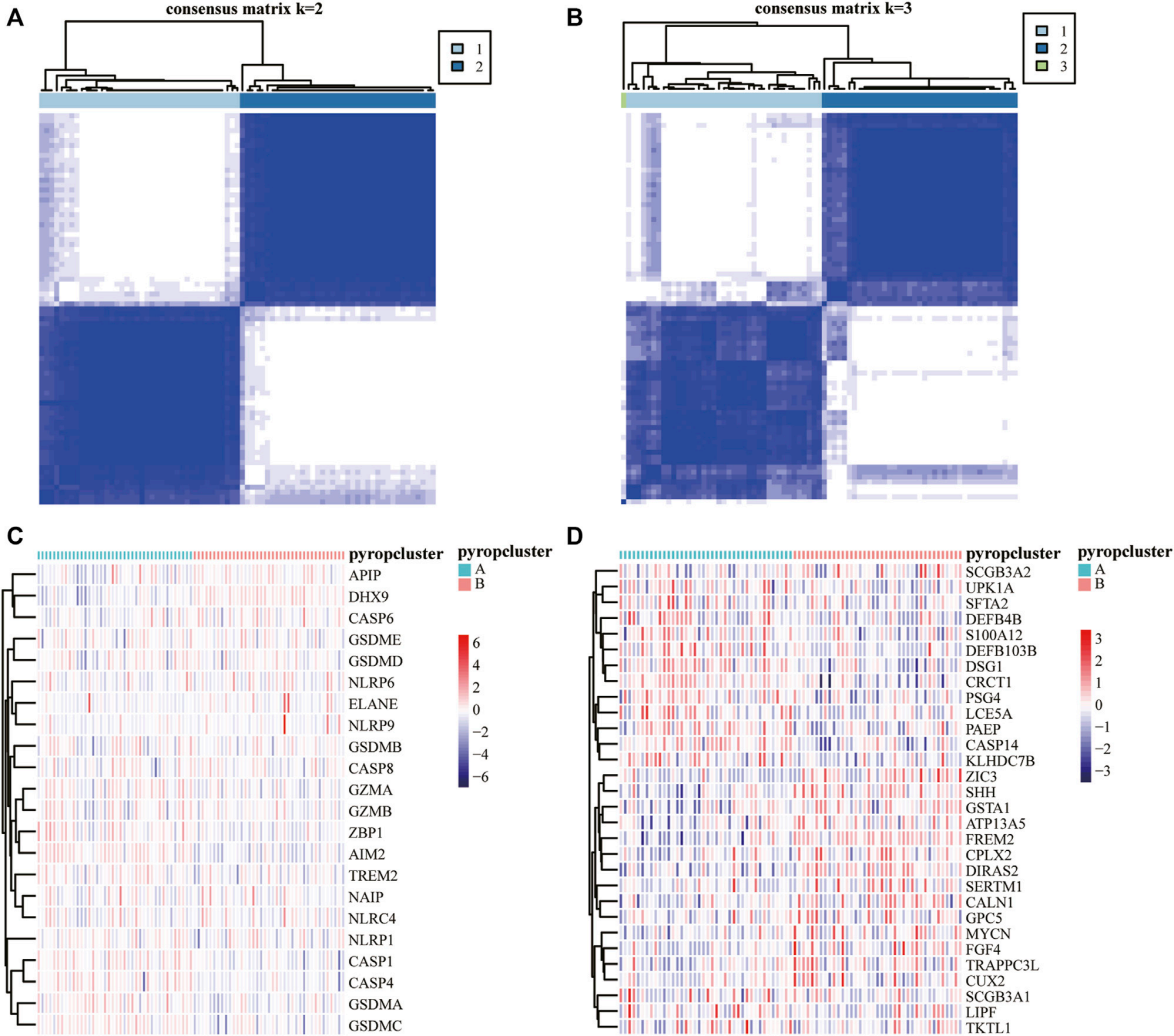
Analysis of pyroptosis subtypes. **(A,B)** Cluster analysis of TCGA-ESCC samples and k = 2 was the most appropriate. **(C)** Expression heatmap of 22 pyroptosis genes in different pyroptosis subtypes. **(D)** Expression heatmap of part of the DEGs between pyroptosis subtypes, red indicates upregulated, blue indicates downregulated.

In order to demonstrate the effect of pyroptosis genes on ESCC, cluster analysis was performed and the TCGA-ESCC samples can be clearly distinguished when they were clustered into two clusters according to the expression difference of pyroptosis genes (Figures 3A, B), and the expression of pyroptosis genes in different subtypes were exhibited by a heatmap (Figure 3C). Then, we used the DESeq2 R package to perform differential expression analysis between the two pyroptosis subtypes to obtain DEGs. Genes with logFC > 1 and adj.*p* < .05 were upregulated genes, and genes with logFC < −1 and adj.*p* < .05 were downregulated genes, finally, 802 upregulated genes and 605 downregulated genes were obtained (Supplementary Figure S1). The expression of some of the DEGs were shown by a heatmap (Figure 3D **)**, and the DEGs were used for subsequent analysis.

### 3.3 Functional enrichment analysis of DEGs

**FIGURE 4:**
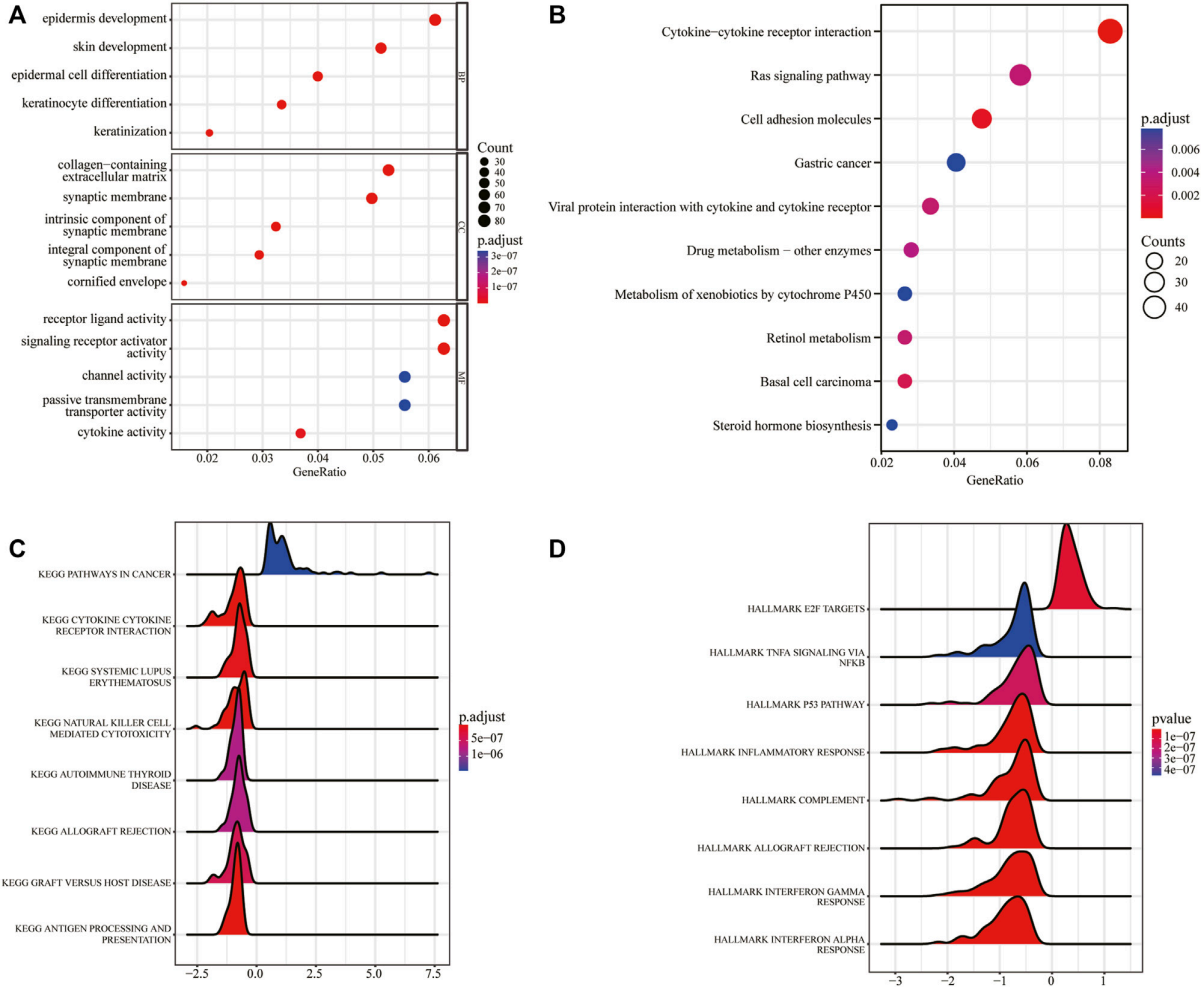
GO/KEGG/GSEA analysis based on DEGs between the pyroptosis subtypes. **(A)** GO functional enrichment analysis. **(B)** KEGG pathway enrichment analysis. **(C)** Gene set enrichment analysis, *x*-axis represents gene ratio, *y*-axis represents KEGG pathways, and the color represents the *p*-value. **(D)** Gene set enrichment analysis, *x*-axis represents gene ratio, the *y*-axis represents HALLMARKs, and the color represents *p*-value.

To examine the function of the DEGs, GO analysis including BP, CC, and MF was performed (Supplementary Table S1). The DEGs were mainly enriched in “epidermis development,” “skin development,” “epidermal cell differentiation” and other BP. At the same time, the cells were enriched in “collagen-containing extracellular matrix,” “synaptic membrane,” “intrinsic component of synaptic membrane” and other CC, and “receptor ligand activity,” “signaling receptor activator activity” and other MF (Figure 4A). Next, KEGG pathways enrichment analysis was also conducted (Supplementary Table S2). The results showed that the DEGs were mainly enriched in biological pathways such as “cytokine-cytokine receptor interaction,” “Ras signaling pathway,” and “cell adhesion molecules” (Figure 4B). To better determine the mechanisms of DEGs in ESCC, GSEA (Supplementary Table S3) was carried out and the results showed that the DEGs mainly affected “pathways in cancer,” “cytokine-cytokine receptor interaction,” “natural killer cell mediated cytotoxicity,” “autoimmune thyroid disease” and other pathways (Figure 4C), and other related functions such as “TNFA signaling *via* NFKB,” “P53 pathway,” “inflammatory response” (Figure 4D).

### 3.4 GSVA based on DEGs in ESCC

**FIGURE 5:**
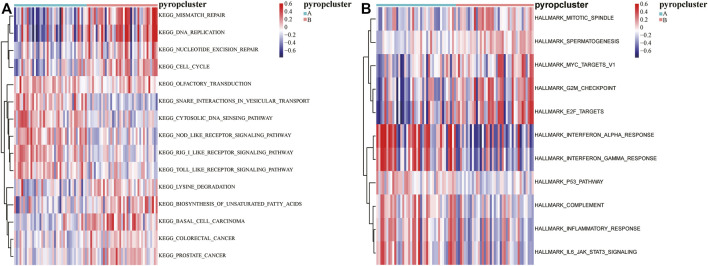
GSVA based on DEGs between the pyroptosis subtypes. **(A)** The enrichment of KEGG pathways in GSVA, red indicates activation and blue indicates inhibition. **(B)** The enrichment of HALLMARKs in GSVA, red indicates activation and blue indicates inhibition.

In order to further explored the function of DEGs, GSVA was executed (Supplementary Table S4), and the results were visualized by heatmaps (Figures 5A, B). We found that there were significant different pathways enriched in the two pyroptosis subtypes. Moreover, some functions and pathways such as “E2F targets,” “P53 pathway,” “inflammatory response,” which further confirmed the results of GSEA, indicating that pyroptosis may affect the activation of these functions and pathways in ESCC.

### 3.5 Establishment of a pyroptosis scoring model

**FIGURE 6:**
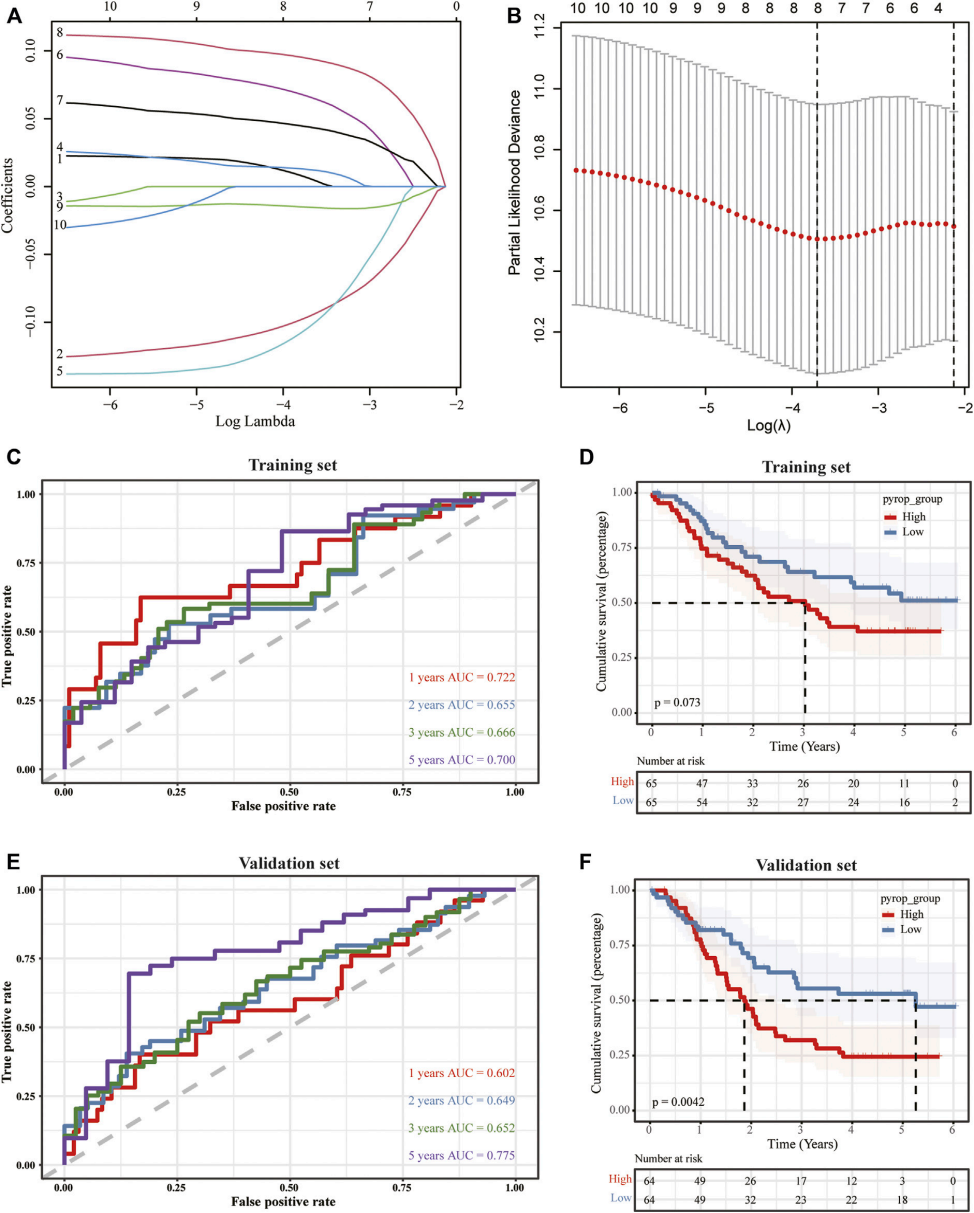
The construction of a pyroptosis scoring model. **(A,B)** Lasso analysis was performed to obtain the pyroptosis-related genes for the construction of the pyroptosis scoring model. **(C)** TimeROC curves of the pyroptosis scoring model in the training set. **(D)** The impact of the pyroptosis scoring model on OS in the training set. **(E)** TimeROC curves against the pyroptosis scoring model in the validation set. **(F)** Survival analysis of the pyroptosis scoring model in the validation set.

To further clarify the effect of pyroptosis on ESCC, we merged the expression profiles of TCGA-ESCC with GSE53625, and the graphs of principal component analysis (PCA) before and after removing batch effect were shown in Supplementary Figures S2A, B, suggesting that the data mixed well after removing the batch effects. Next, we randomly divided the merged dataset into a training set and a validation set with a ratio of 1:1. Then, univariate Cox regression analysis was performed to calculate the association between the DEGs and OS, and the genes with *p* < .05 were selected to the Lasso regression analysis in the training set (Figures 6A, B). Finally, the pyroptosis-related genes were obtained and a pyroptosis scoring model was built, pyroptosis score = ADGRG2 × .006225234 + CARD18 × (−.095822011) + FGD5 × .012532728 + FSTL4 × (−.103861554) + FXYD5 × .066290533 + MAMDC2 × .043936646 + PCSK2 × .094385321 + PLA2G4E × (−.015183865), and the patients were divided into high pyrop_group and low pyrop_group according to the median of pyroptosis score. After building the model, the timeROC curves and the AUC values were used to evaluate the prognostic predictive accuracy at 1-year, 2-year, 3-year, and 5-year. The results showed that the AUC of the model in the training set was .722 at 1-year, .655 at 2-year, .666 at 3-year, and .700 at 5-year (Figure 6C). Furthermore, Kaplan-Meier analysis showed that the curves for OS between the high pyrop_group and low pyrop_group in the training set could be well distinguished (Figure 6D). In the validation set, the 1-year AUC value was .602, the 2-year AUC value was .649, the 3-year AUC value was .652, and the 5-year AUC value was .775 (Figure 6E), more importantly, there was a significant difference on OS between the high pyrop_group and low pyrop_group, and the high pyrop_group was associated with worse OS (Figure 6F), indicating that the model could accurately predict the prognosis of ESCC patients, especially the assessment of long-term survival rate.

### 3.6 Pyroptosis scoring was associated with the prognosis of ESCC

**FIGURE 7:**
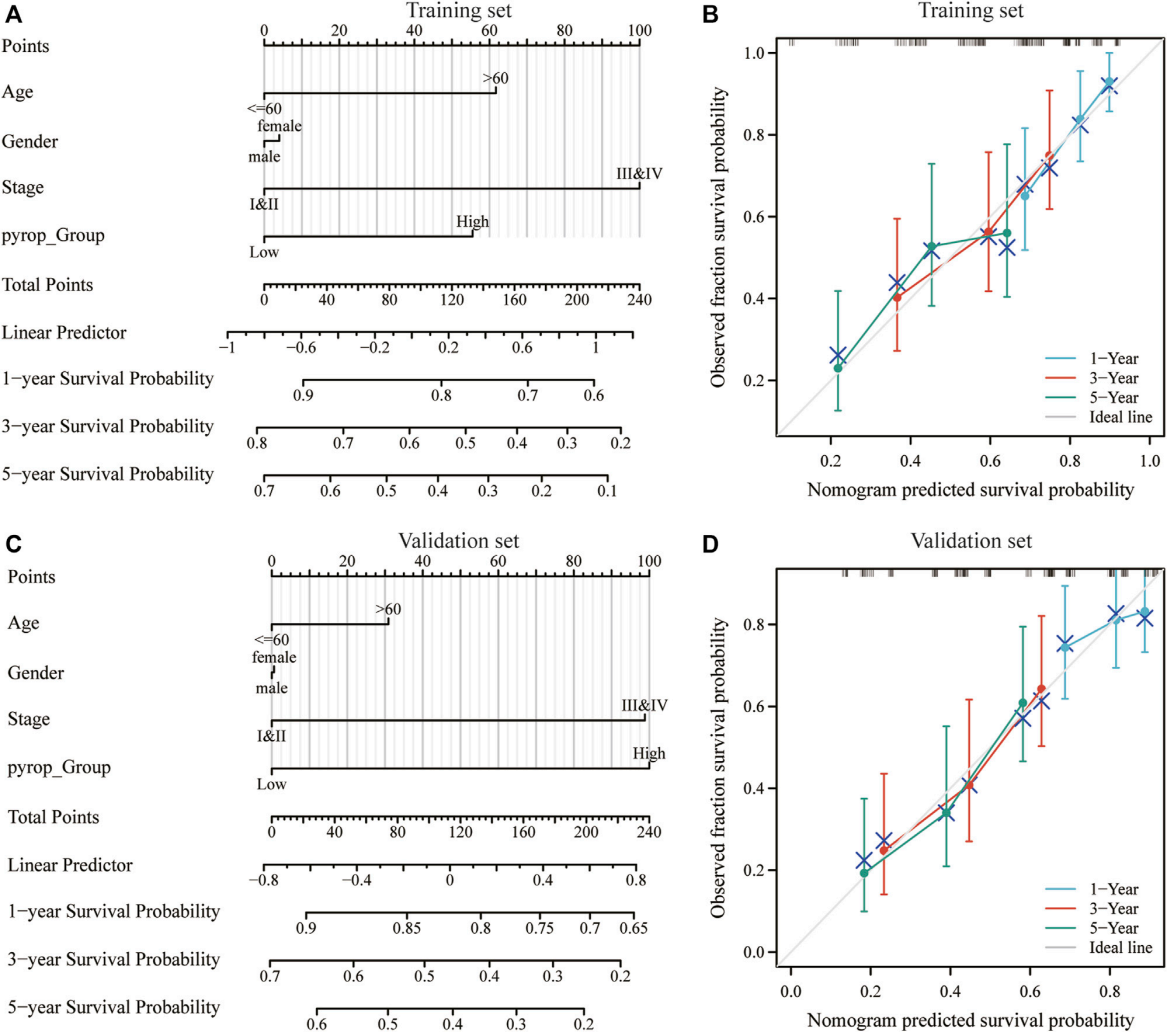
Effect of pyroptosis scoring on the prognosis of ESCC patients. **(A)** Nomogram model included the pyroptosis scoring in the training set. **(B)** Calibration curves at 1-year, 3-year, and 5-year in the training set. **(C)** Nomogram model included the pyroptosis scoring in the validation set. **(D)** Calibration curves at 1-year, 3-year, and 5-year in the validation set. *p < .05; **p < .01; ***p < .001.

To further clarify the effect of pyroptosis scoring model on the prognosis of ESCC, on the basis of univariate and multivariate Cox regression analysis (Supplementary Tables S5, S6), nomogram models included the pyroptosis scoring was established in the training set and validation set, and the pyroptosis scoring was found to have a great contribution to the prognosis (Figures 7A, C). Meanwhile, the calibration curves at 1-year, 3-year, and 5-year in the training set and validation set were close to the ideal line, indicating that the nomogram models have a good prognostic predictive performance on ESCC (Figures 7B, D).

### 3.7 Correlation between pyroptosis scoring and immune cell infiltration

**FIGURE 8:**
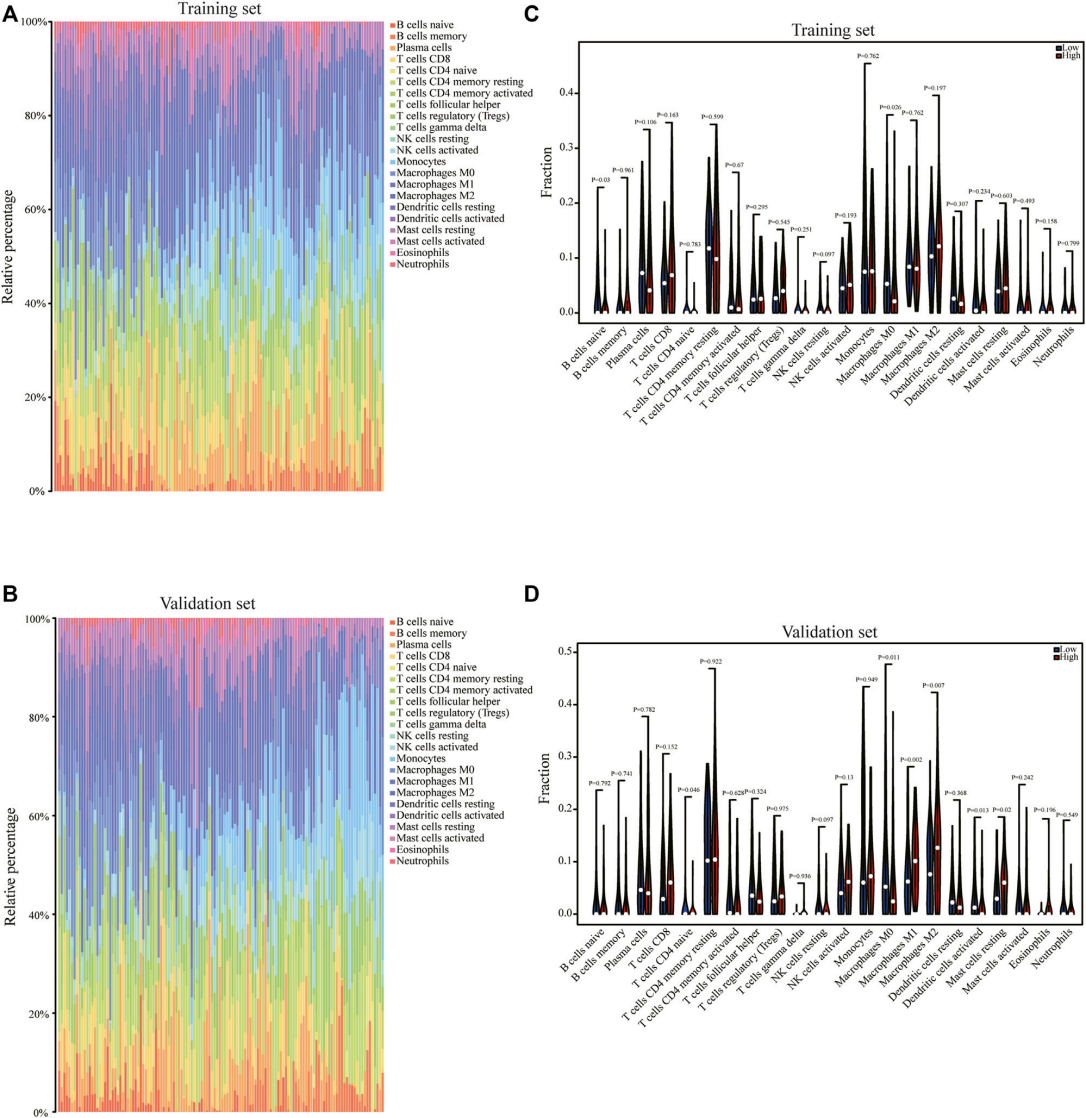
The correlation of pyroptosis scoring and immune cell infiltration. **(A)** The CIBERSORTx algorithm was used to analyze the immune cell infiltration of each sample in the training set. **(B)** The CIBERSORTx algorithm was used to analyze the immune cell infiltration of each sample in the validation set. **(C)** Relationship between the pyroptosis scoring and the expression of immune cells in the training set. **(D)** Relationship between the pyroptosis scoring and the abundance of immune cells in the validation set.

**FIGURE 9:**
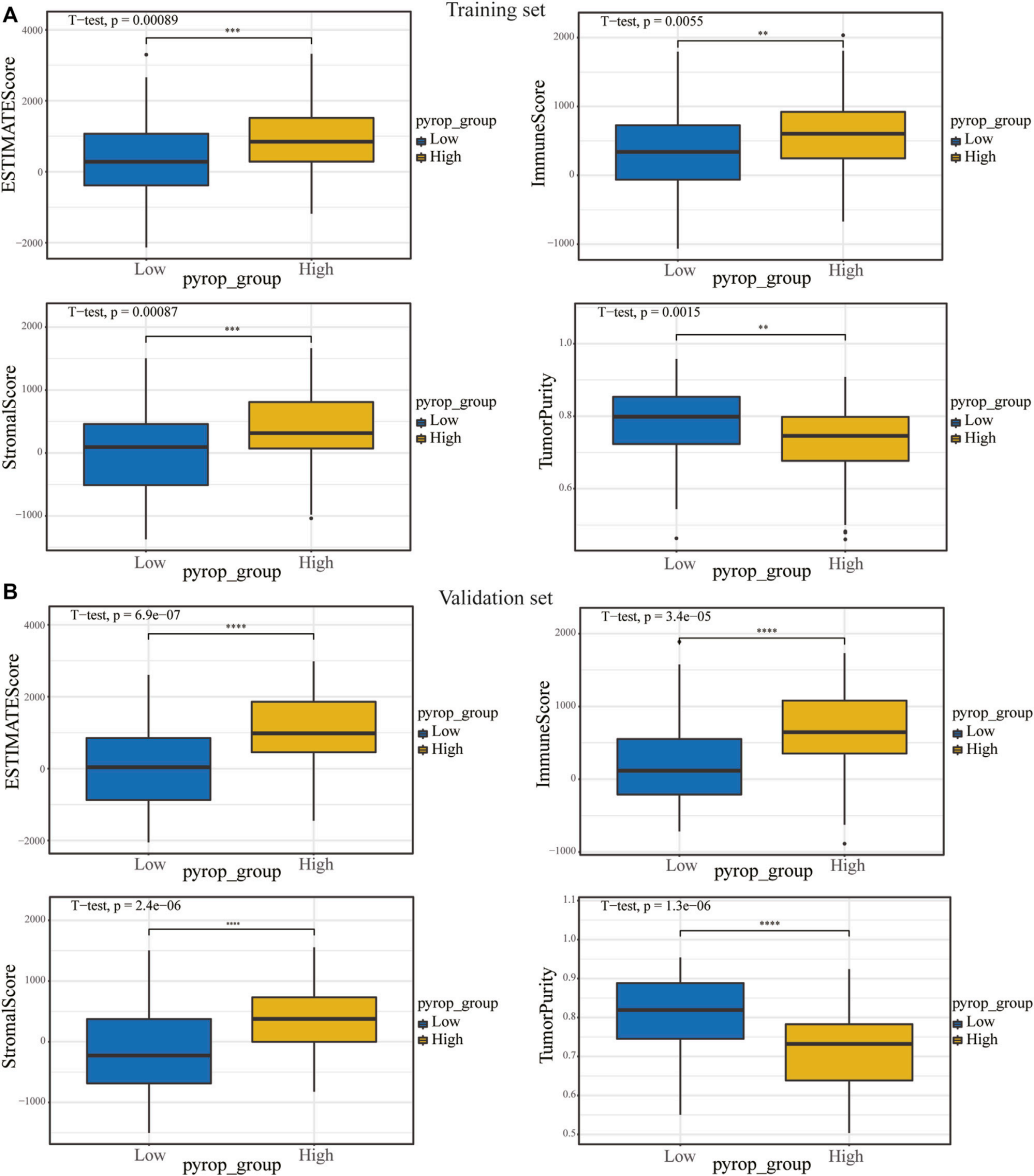
The relationship between pyroptosis scoring and immune score. **(A)** In the training set, the effect of pyroptosis scoring on ESTIMATEScore, ImmuneScore, StromalScore, and TumorPurity. **(B)** Differences in ESTIMATEScore, ImmuneScore, StromalScore and TumorPurity between different pyroptosis scoring groups in the validation set. **p < .01; ***p < .001; ****p < .0001.

In order to analyze the correlation between the pyroptosis scoring model and immune cell infiltration, we used the CIBERSORTx algorithm to calculate the immune infiltration level of 22 immune cells in each sample in the training set and validation set, respectively (Figures 8A, B). The expression differences of 22 types immune cells between the high pyrop_group and low pyrop_group in training set and validation set were performed, respectively (Figures 8C, D). The results showed that the expression of M0 macrophages were lower in the high pyrop_group than in the low pyrop_group in the training set and validation set, while the expression of M1 and M2 macrophages in the high pyrop_group were higher than those in the low pyrop_group in the validation set, which suggested that pyroptosis-related genes can affect the activation pathways of macrophages and the immune microenvironment in ESCC. In addition, the ESTIMATE algorithm was carried out to assess the correlation between pyroptosis scoring and immune cell infiltration as well. In the training set (Figure 9A), the ESTIMATEScore, ImmuneScore, and StromalScore in the high pyrop_group were higher than the low pyrop_group, while the TumorPurity was opposite. Similarly, in the validation set (Figure 9B), the results of the ESTIMATEScore, ImmuneScore, StromalScore, and tumor purity were all consistent with the training set.

## 4 Discussion

ESCC is a deadly malignant tumor and the curative effects of various treatment methods are not satisfactory, what’s worse is that there is lack of effective targeted therapy drugs at present. Therefore, the treatment of ESCC is a huge challenge for clinicians, and it is urgent to explore more effective treatment strategies.

In this study, we found that a novel pyroptosis scoring model constructed by pyroptosis-related genes was associated with the prognosis of ESCC, which was similar to the previous literature reporting that pyroptosis-related genes were associated with the prognosis of EAC (Zeng et al., 2021; Li et al., 2022a). At the same time, our study suggested that a high pyroptosis score was associated with a poorer OS in ESCC. One possible explanation is that pyroptosis is a way of pro-inflammatory cell death, and a high pyroptosis score provides a suitable microenvironment for tumor growth and promotes tumor growth, leading to worse prognosis, this effect of pyroptosis has been confirmed in previous studies (Fang et al., 2020; Yu et al., 2021; Huang et al., 2022).Therefore, the pyroptosis scoring model constructed by us for the first time indicate that pyroptosis-related genes were involved in the biological process of ESCC to some extent and affect the prognosis of ESCC patients.

As we all known, the occurrence and development of tumors are closely related to the changes of tumor microenvironment (Gajewski et al., 2013; Fu and Jiang, 2018; Lian et al., 2020), and ESCC is no exception (Zheng et al., 2020), more importantly, immunotherapy has achieved encouraging results in the clinical trials of ESCC (Kelly et al., 2021; Doki et al., 2022).Therefore, in-depth study of ESCC immune microenvironment is a key step to improve the prognosis of ESCC. Here, our study found that pyroptosis scoring model was involved in the expression of macrophages in ESCC, suggesting that the regulation of macrophage polarization may be an effective anti-tumor strategy, which was partly consistent with the previous finding that reprogram tumor-associated macrophages to achieve anti-tumor function (Zhang et al., 2019). We all know that M0 macrophages are dormant macrophages, which can be induced to be polarized into M1 macrophages or M2 macrophages. M1 macrophages are generally activated by interferon-γ and lipopolysaccharide, secreting proinflammatory factors and plays an important role in the early stage of inflammation. M2 macrophages play a vital role in angiogenesis, secretion of anti-inflammatory factors, and promotion of tissue repair and wound healing. Meanwhile, M2 macrophages can produce various matrix metalloproteinases (MMPs) and chemokines, such as MMP-2, MMP-7, MMP-9, CCL18, and CCL22, which promote the metastasis of cancer cells. Therefore, the changes of macrophages in ESCC can affect the immune microenvironment of ESCC, resulting in complex changes in the pathophysiological process of ESCC. Collectively, pyroptosis phenotype and tumor microenvironment interact with each other, and ultimately affect the prognosis of ESCC. Thus, immunotherapy targeting macrophage polarization may be a hopeful immunotherapeutic method for ESCC intervention.

In addition, GSEA and GSVA were enriched in “pathways in cancer,” “P53 pathway,” “mismatch repair,” “DNA replication,” “cell cycle,” “G2M checkpoint” and other signaling pathways, suggesting that pyroptosis-related genes may also affect tumor development process by affecting tumor cell cycle and cell proliferation. However, few studies of pyroptosis have reached similar conclusion to ours, which means that we may have made some new discoveries and more studies are needed to confirm them in the future.

Here are the highlights of our research. Firstly, the GSE53625 with the largest ESCC sample size in the GEO and all the TCGA-ESCC samples were included in this study, and the classification of pyroptosis subtypes of ESCC was performed for the first time. Based on pyroptosis subtypes classification, a pyroptosis scoring model was established, which was found to be related to the prognosis and immune microenvironment in ESCC. Secondly, how to improve the prognosis of ESCC is a huge challenge for medical researchers, this study revealed the relationship between pyroptosis-related genes and ESCC, improving our understanding of ESCC, providing a new insight and potential treatment strategy for ESCC. Finally, the classification of pyroptosis is conducive to identify the genetic differences of different ESCC patients, which is greatly helpful to implement the precise individualized treatment for different patients.

Although our study has increased our understanding of ESCC, there are still some limitations. Firstly, this study was mainly based on bioinformatics analysis, and the conclusion obtained need to be confirmed by *in vivo* and *in vitro* experiments. Secondly, the samples size was relatively small, in order to improve the credibility of the research, it is necessary to expand the sample size in further research in the future.

In conclusion, our study revealed that a pyroptosis scoring model was related to the prognosis and immune microenvironment of ESCC, which provides a novel insight and a hopeful treatment strategy for ESCC.

## Data Availability

The datasets presented in this study can be found in online repositories. The names of the repository/repositories and accession number(s) can be found in the article/Supplementary Material.
