# Supplementary material for: A novel pyroptosis scoring model was associated with the prognosis and immune microenvironment of esophageal squamous cell carcinoma
Source: Front Genet. 2023 Jan 4;13:1034606. doi: 10.3389/fgene.2022.1034606 (PMC9845255; doi:10.3389/fgene.2022.1034606)
Supplement: Supplementary file 1 [file DataSheet1.PDF]

Supplementary Material

1 Supplementary Tables

Table S1. GO enrichment analysis of DEGs

Ontology	ID	Description	p.adjust
BP	GO:0008544	epidermis development	1.50E-18
BP	GO:0043588	skin development	2.92E-16
BP	GO:0030216	keratinocyte differentiation	2.27E-13
BP	GO:0009913	epidermal cell differentiation	1.13E-12
BP	GO:0031424	keratinization	6.58E-12
CC	GO:0099240	intrinsic component of synaptic membrane	3.70E-12
CC	GO:0099699	integral component of synaptic membrane	4.67E-11
CC	GO:0001533	cornified envelope	4.67E-11
CC	GO:0097060	synaptic membrane	3.88E-10
CC	GO:0062023	collagen-containing extracellular matrix	5.30E-10
MF	GO:0048018	receptor ligand activity	5.60E-10
MF	GO:0030546	signaling receptor activator activity	6.74E-10
MF	GO:0005125	cytokine activity	1.75E-08
MF	GO:0015267	channel activity	3.40E-07

MF	GO:0022803	passive transmembrane transporter activity	3.40E-07

Table S2. KEGG enrichment analysis of DEGs

ID	Description	p.adjust
hsa04060	Cytokine-cytokine receptor interaction	2.041E-05
hsa04514	Cell adhesion molecules	0.000587512
hsa05217	Basal cell carcinoma	0.002096726
hsa04061	Viral protein interaction with cytokine and cytokine receptor	0.003291542
hsa00830	Retinol metabolism	0.003291542
hsa04014	Ras signaling pathway	0.003480718
hsa00983	Drug metabolism - other enzymes	0.004007286
hsa00980	Metabolism of xenobiotics by cytochrome P450	0.007800032
hsa00140	Steroid hormone biosynthesis	0.007800032
hsa05226	Gastric cancer	0.007800032

Table S3. Gene set enrichment analysis of DEGs

Description	NES	p.adjust
KEGG_ANTIGEN_PROCESSING_AND_PRESENTATION	-2.636619769	8.12E-09
KEGG_CYTOKINE_CYTOKINE_RECEPTOR_INTERACTION	-2.085046847	8.12E-09

KEGG_NATURAL_KILLER_CELL_MEDIATED_CYTOTOXICITY	-2.316690348	8.12E-09
KEGG_SYSTEMIC_LUPUS_ERYTHEMATOSUS	-2.270473063	5.24E-08
KEGG_GRAFT_VERSUS_HOST_DISEASE	-2.447539354	4.20E-07
KEGG_ALLOGRAFT_REJECTION	-2.436958102	7.93E-07
KEGG_AUTOIMMUNE_THYROID_DISEASE	-2.414225663	7.94E-07
KEGG_PATHWAYS_IN_CANCER	1.816424179	1.45E-06
HALLMARK_ALLOGRAFT_REJECTION	-2.554954123	1.25E-09
HALLMARK_INFLAMMATORY_RESPONSE	-2.136076385	1.25E-09
HALLMARK_INTERFERON_ALPHA_RESPONSE	-2.933577352	1.25E-09
HALLMARK_INTERFERON_GAMMA_RESPONSE	-2.803476501	1.25E-09
HALLMARK_COMPLEMENT	-2.159191525	1.51E-09
HALLMARK_E2F_TARGETS	1.928974438	5.43E-07
HALLMARK_P53_PATHWAY	-1.908407356	1.17E-06
HALLMARK_TNFA_SIGNALING_VIA_NFKB	-1.855180072	2.99E-06

Table S4. Gene Set Variation Analysis of DEGs

ID	logFC	p.adjust
KEGG_RIG_I_LIKE_RECEPTOR_SIGNALING_PATHWAY	0.118725748	0.004969941
KEGG_LYSINE_DEGRADATION	-0.130653834	0.004969941
KEGG_MISMATCH_REPAIR	-0.201112705	0.004969941

KEGG_CYTOSOLIC_DNA_SENSING_PATHWAY	0.11391924	0.009354038
KEGG_BASAL_CELL_CARCINOMA	-0.11920621	0.009668961
HALLMARK_G2M_CHECKPOINT	-0.157350897	0.030010679
HALLMARK_MITOTIC_SPINDLE	-0.104397522	0.041486711
HALLMARK_COMPLEMENT	0.093242496	0.044759049
HALLMARK_INFLAMMATORY_RESPONSE	0.100545313	0.069418666
HALLMARK_MYC_TARGETS_V1	-0.107731254	0.069418666

Table S5. Univariate and multivariate Cox regression analysis of factors associated with OS in the training set

Characteristics	Total(N)	Univariate analysis		Multivariate analysis	
		Hazard ratio (95% CI)	p value	Hazard ratio (95% CI)	p value
**Age**	130				
>60	48	Reference			
<=60	82	0.650 (0.389-1.087)	0.101		
**Gender**	130				
female	23	Reference			
male	107	0.914 (0.475-1.757)	0.786		
**Stage**	130				
I&II	74	Reference			

III&IV	56	2.380 (1.422-3.983)	**<0.001**	2.299 (1.371-3.855)	**0.002**
**pyrop_group**	130				
Low	65	Reference			
High	65	1.600 (0.954-2.683)	0.075	1.490 (0.886-2.504)	0.132

Table S6. Univariate and multivariate Cox regression analysis of factors associated with OS in the validation set

Characteristics	Total(N)	Univariate analysis		Multivariate analysis	
		Hazard ratio (95% CI)	p value	Hazard ratio (95% CI)	p value
**Age**	128				
>60	64	Reference			
<=60	64	0.644 (0.399-1.039)	0.071	0.811 (0.497-1.325)	0.404
**Gender**	128				
female	22	Reference			
male	106	1.172 (0.629-2.186)	0.617		
**Stage**	127				
I&II	66	Reference			
III&IV	61	2.062 (1.258-3.381)	**0.004**	1.949 (1.185-3.207)	**0.009**
**pyrop_group**	128				
Low	64	Reference			
High	64	2.029 (1.238-3.325)	**0.005**	1.966 (1.182-3.269)	**0.009**

2 Supplementary Figures

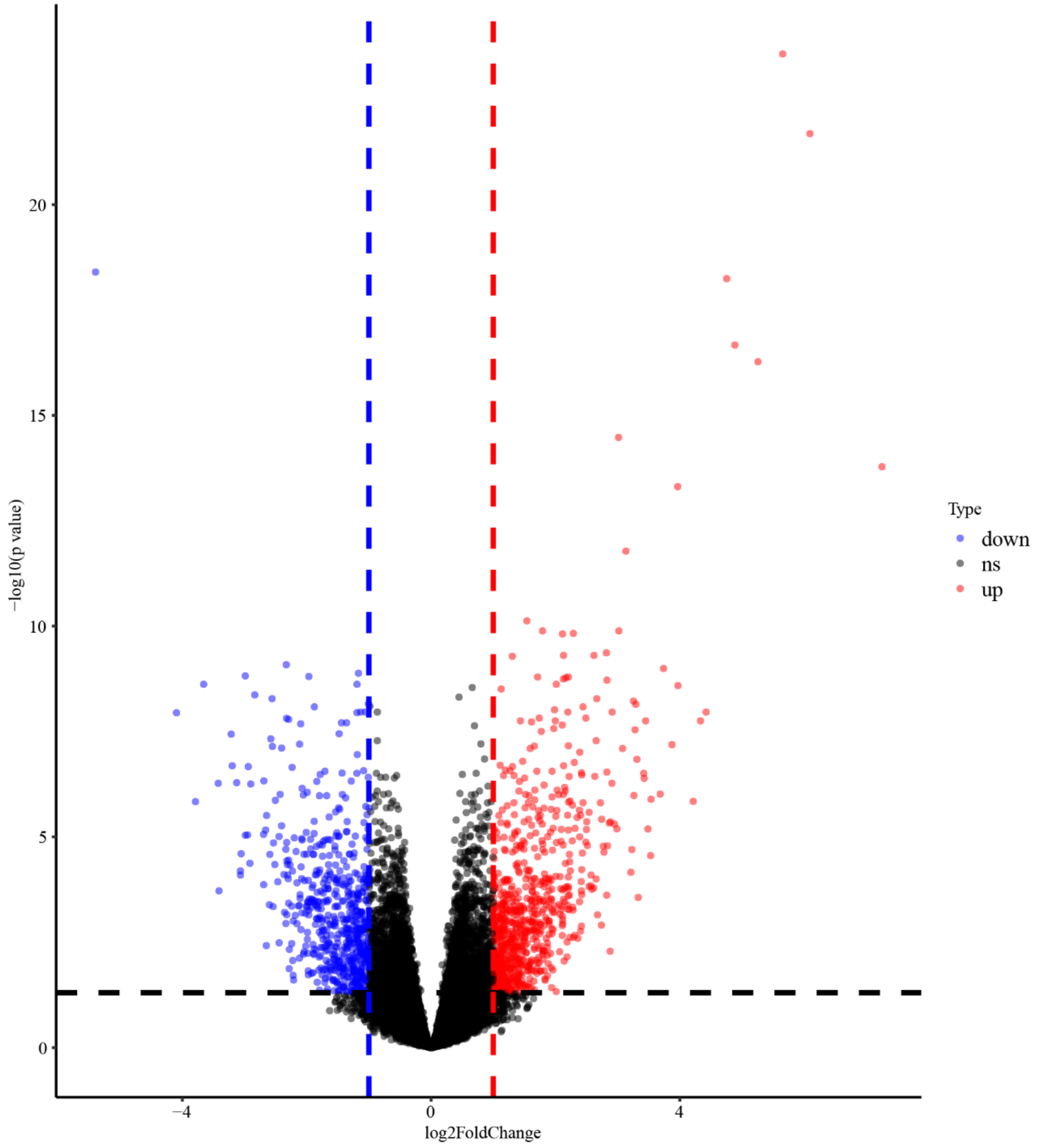

Supplementary Figure 1. Volcano plot of the DEGs. Genes from differential expression analysis of samples from different pyroptosis subtypes were visualized by a volcano plot.

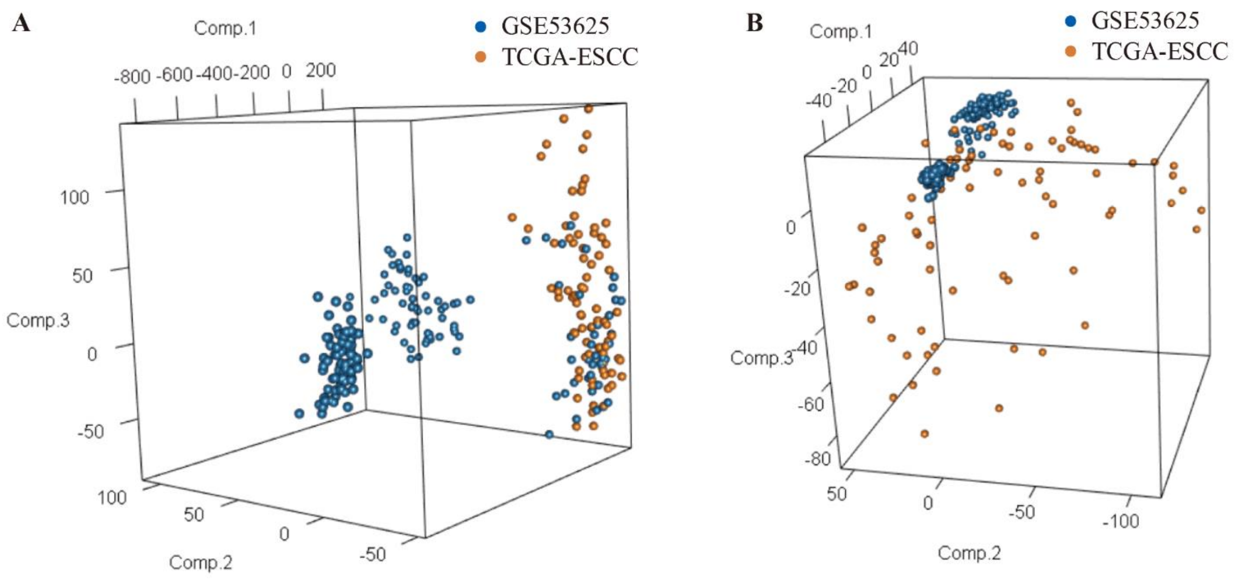

Supplementary Figure 2. PCA analysis before and after the combination of TCGA-ESCC and GSE53625. (A) PCA analysis plot before data merging. **(B)** PCA plot of combined data after removing the batch effects.
